# Analysis of intensive care unit admissions for older adults with femoral fractures: a retrospective cohort

**DOI:** 10.1590/1980-220X-REEUSP-2023-0398en

**Published:** 2024-08-02

**Authors:** Carolina Rodrigues Mendes Nogueira Cobra, Paulo Carlos Garcia, Isadora Castilho Moreira de Oliveira Passos, Greiciane da Silva Rocha, Lilia de Souza Nogueira

**Affiliations:** 1 Universidade de São Paulo, Escola de Enfermagem, São Paulo, SP, Brazil.; 2 Universidade de São Paulo, Hospital Universitário, São Paulo, SP, Brazil.; 3 Universidade Federal do Acre, Centro de Ciências da Saúde e do Desporto, Rio Branco, AC, Brazil.

**Keywords:** Femoral Fractures, Aged, Intensive Care Units, Workload, Mortality, Fracturas del Fémur, Anciano, Unidades de Cuidados Intensivos, Carga de Trabajo, Mortalidad

## Abstract

**Objectives:**

To describe the historical series of admissions to the Intensive Care Unit of older adults with femoral fractures, and verify the association between age and injury characteristics and treatment, nursing workload, severity, and clinical evolution in the unit.

**Method:**

Retrospective cohort of 295 older adults (age ≥60 years) admitted to the Intensive Care Unit of a hospital in São Paulo, between 2013 and 2019, and who presented with a femur fracture as the main cause of hospitalization. Variables regarding demographic characteristics, cause, and type of fracture, treatment provided, severity, nursing workload, and medical outcome of patients were analyzed. The Shapiro-Wilk, Wilcoxon-Mann-Whitney, Kruskal-Wallis tests and Pearson correlation were applied.

**Results:**

There was an increase in older adults admission to the Intensive Care Unit from 2017 on. Female patients with distal femur fractures who died in the Intensive Care Unit had significantly (p < 0.05) higher median age than men, patients with shaft or proximal femur fractures, and survivors.

**Conclusion:**

The study findings highlight essential information for structuring care for older adults with femoral fractures who require intensive care.

## RESUMEN

**Objetivo:**

Describir la serie histórica de internaciones, en la Unidad de Cuidados Intensivos, de ancianos con fractura de fémur, y verificar la asociación entre la edad y las características y tratamiento de la lesión, carga de trabajo de enfermería, gravedad y evolución clínica en la unidad.

**Método:**

Cohorte retrospectiva de 295 ancianos (edad ≥60 años) ingresados en la Unidad de Cuidados Intensivos de un hospital de São Paulo, entre 2013 y 2019, y que presentaron fractura de fémur como principal causa de hospitalización. Se analizaron variables relativas a características demográficas, causa y tipo de fractura, tratamiento realizado, gravedad, carga de trabajo de enfermería y evolución clínica de los pacientes. Se aplicaron las pruebas de *Shapiro-Wilk, Wilcoxon-Mann-Whitney*, *Kruskal-Wallis* y correlación de *Pearson*.

**Resultados:**

Hubo aumento de hospitalizaciones de ancianos en Unidad de Cuidados Intensivos desde 2017. Las pacientes del sexo femenino con fracturas de fémur distal que murieron en la Unidad de Cuidados Intensivos tuvieron mediana de edad significativamente (p < 0,05) más alta que los hombres, los pacientes con fracturas de diáfisis o de fémur proximal y los supervivientes.

**Conclusión:**

Los hallazgos del estudio resaltan información esencial para estructurar la atención de los ancianos con fracturas femorales que requieren tratamiento intensivo.

## INTRODUCTION

Since the 1970s, there has been a sociodemographic transition in Brazil with a significant increase in the proportion of older adults compared to any other age group. According to the Brazilian Institute of Geography and Statistics (*IBGE*), the country will experience significant population growth of older adults by 2039 and, in 2050, this group will surpass that of children, turning Brazil into one of the most aged countries in the world^(1)^.

The aging process is physiologically characterized by a reduction in muscle mass and organic and functional loss that directly interfere with greater dependence and need for care, as well as changes in balance and gait deficits, which can increase the risk of falls and, consequently, the occurrence of femur fractures in the older adults^(2,3)^. A study shows that, besides advanced age, variables related to the female sex, low body mass index, presence of osteoporosis, greater length and lower average thigh circumference are risk factors for the occurrence of femur fractures in the older population^(4)^.

Femur fractures can occur in the proximal, distal region or in the femoral shaft^(5)^. Femur fractures in the proximal region are the most common and result mainly from falls from standing height in people over 60 years of age^(6)^. The impact of femoral fractures on the older adults’ health conditions is evident, as this type of injury contributes to increased fragility and dependence, reducing quality of life^(7,8)^. Regarding the clinical outcome, a time series study showed that, between 2008 and 2018, the average mortality rate of older adults due to femur fractures was 5%, being higher among those aged over 80 years^(9)^.

Therefore, this type of fracture, prevalent in older individuals, leads to hospitalization and surgical treatment, representing a high social and economic cost due to the prolonged hospital stay and rehabilitation periods^(9,10)^. Furthermore, the occurrence of complications in the postoperative period after femoral fracture correction, often associated with the presence of comorbidities and the vulnerability of the older adults, highlights the need for continuous surveillance in the Intensive Care Unit (ICU) focused on reducing complications and deaths^(7,8,10)^.

In this regard, the nurse’s role alongside the multidisciplinary team in caring for older adults with femoral fractures admitted to the ICU is essential and includes actions to prevent complications, such as healthcare-associated infections (HAIs) and pressure injury, pain control, psychological care, rehabilitation as early as possible, among others^(11)^. Still regarding patient care in the ICU, researchers found that the nursing workload required by older adults during their length of stay in the critical care unit is higher compared to adults^(12)^, thus emphasizing the importance of analyzing the care demand of this population for quantitative and qualitative adaptation of human resources focused on ensuring quality of care.

Although the study cited above^(12)^ highlights the high nursing workload of older adults in the ICU, the sample analyzed consisted of patients with different pathologies, which may not reflect the characteristics of such a specific population, such as older adults with femoral fractures who require intensive care. Furthermore, there is a knowledge gap in the literature about the possible association between the age of this population and variables related to the characteristics of the injury, treatment provided to correct the femur fracture, severity, and outcome in the ICU.

In view of this, the performance of this study is warranted, the results of which may contribute, in clinical practice, to the recognition of possible geriatric characteristics and needs that can help promote a better nursing process aimed at the older adults, so as to focus on diagnoses, outcomes, and priority interventions, training of professionals to assist older adults with femoral fractures admitted to the ICU, and better management of human resources and of the quality of care provided, to minimize complications and reduce professional burnout. Thus, the present study has the objective to describe the historical series of admissions of older adults with femoral fractures to the ICU, and verify the association between age and the characteristics and treatment of the injury, nursing workload, severity, and clinical evolution in the unit.

## METHOD

### Design of Study

This is a retrospective cohort study that analyzed medical records of older patients (≥ 60 years old) admitted to the ICU.

### Local

The study was carried out in a university hospital located in the city of São Paulo, Brazil. The institution has a general ICU, with 12 beds, which provides assistance to critical medical and surgical patients.

### Population and Selection Criteria

The sample consisted of older adults admitted to the ICU between January 1, 2013 and December 31, 2019, and who presented with a femur fracture as the cause of hospital admission and a main injury, characterized by the absence of injury, according to the Abbreviated Injury Scale (AIS*)* ^(13)^, greater than or equal to 3 in another body region.

The AIS is a scale used to describe injuries resulting from trauma and identifies their severity, classifying them into: AIS 1 (minor injury), AIS 2 (moderate injury), AIS 3 (serious injury), AIS 4 (severe injury with imminent life threat), AIS 5 (critical injury, with uncertain survival) or AIS 6 (injury of maximum severity, incompatible with life)^(13)^. Femur fracture is classified as an AIS 3 injury, according to the AIS manual^(13)^.

### Variables

The variables analyzed in the study were age, sex, cause of the femur fracture (fall from own height, fall from one level to another or other causes), anatomical region of the fracture (proximal, distal or shaft of the femur), type of treatment (surgical or non-surgical), length of stay (in days), and clinical outcome in the ICU (survivor or non-survivor), Charlson comorbidity index^(14)^, risk of death according to the Simplified Acute Physiology Score version 3 (SAPS 3)^(15)^, and nursing workload measured by the Nursing Activities Score (NAS)^(16)^.

Charlson comorbidity index is a prognostic method which evaluates 19 previous clinical conditions of the patient and, for each of them, assigns a score from zero to six. These points are summed and the index final value reflects the weight of the patient’s comorbidities on mortality, that is, higher index scores are associated with an increased frequency of deaths^(14)^. SAPS 3 is a prognostic score consisting of 20 variables subdivided into demographic, reasons for admission to the ICU, and physiological, which represent the degree of disease impairment and allow defining, through a logistic regression equation, the patient’s risk of death in the ICU^(15)^.

The NAS is an instrument for measuring nursing workload composed of 23 nursing activities distributed into seven major categories (basic activities, ventilatory, cardiovascular, renal, neurological and metabolic support, and specific interventions), with each activity performed assigning a weight in the final score. The NAS allows identifying the percentage of time spent by nursing professionals in direct and indirect patient care in the last 24 hours of ICU admission, reaching a maximum of 176.8^(16)^. Researchers carried out the transcultural adaptation and validation of the NAS for the Portuguese language and found that the instrument presented satisfactory reliability, criterion and construct validity indexes, being considered a valid and reliable indicator to measure the nursing workload in the ICU in Brazil^(17)^.

### Data Collection

The admissions analysis period began in 2013, the year in which the instrument for measuring nursing workload in the study ICU was computerized and ended in December 2019, as, in 2020, there was a significant change in the admissions profile in the ICU due to the COVID-19 pandemic.

For data collection, four instruments were applied: demographic data, related to femoral fracture and clinical evolution in the ICU (instrument 1), calculation of Charlson comorbidity index (instrument 2), NAS score (instrument 3), and SAPS 3 calculation (instrument 4). Information was collected from patients’ physical records and the ICU’s computerized system. For the SAPS 3 index, data from the first hour of the patient’s admission to the ICU were considered and the calculation was carried out in the spreadsheet available on the SAPS 3 public domain website *Research Group* (https://www.saps3.org/archive/downloads/user-agreement/downloads/). The information on NAS admission (referring to the patient’s first 24 hours in the unit) and NAS stay in the ICU (referring to NAS values during the entire stay in the critical unit) were retrieved from the ICU computerized system that is fed daily by nurses in the unit, trained periodically to calculate the score.

### Data Analysis and Treatment

The data were tabulated in the software *Microsoft Excel* ^®^ 2013 and analyzed with the support of a statistics professional using the R software version 4.1.2. To analyze the historical series proposed in the objective of the study, a combined bar and line graph was constructed describing the number of monthly admissions to the ICU of older adults with femoral fractures and the patients’ average age. To verify the association between age and the categorical variables of the study, the Shapiro-Wilk test was first applied, identifying non-normal distribution of the age of patients in the sample. Therefore, Wilcoxon-Mann-Whitney and Kruskal-Wallis nonparametric tests were used in association analyses. Regarding the correlation between age and numerical variables, Pearson correlation coefficient, with no correlation being considered the value of the coefficient of 0.00 to 0.10; weak correlation, from 0.10 to 0.39; moderate correlation, from 0.40 to 0.69; strong correlation, from 0.70 to 0.89; and very strong correlation, from 0.90 to 1.00. In all analyses, a significance level of 5% was established.

### Ethical Aspects

The research was approved in 2021 by the institution’s Research Ethics Committee (CEP) (Opinion No. 4.789.403) and, as it was a retrospective analysis of patient records, the CEP waived the application of the Free and Informed Consent Form to the participants.

## RESULTS

**Figure 1:**
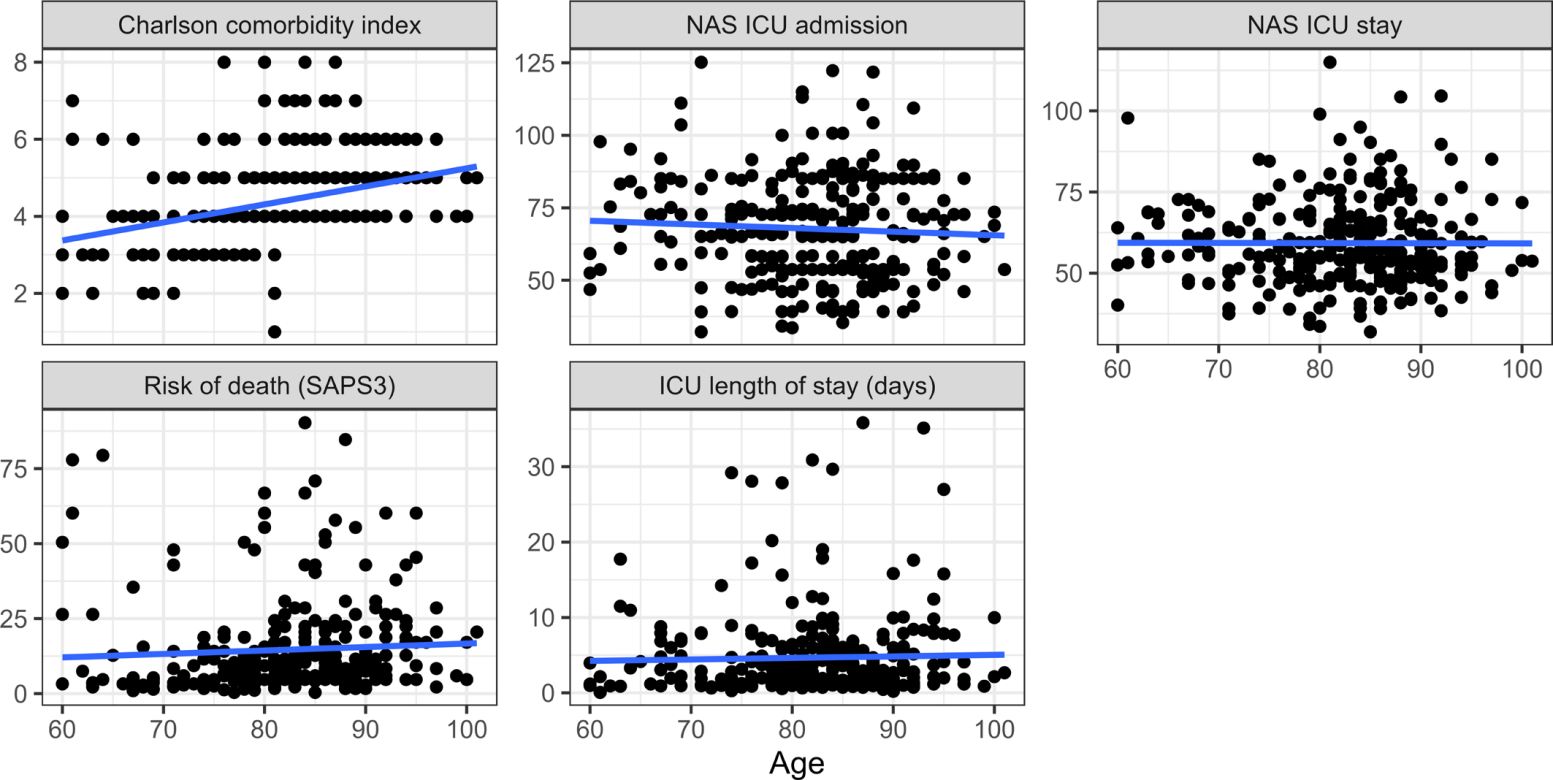
Monthly distribution of ICU admissions of older adults with femoral fractures and average age of patients. São Paulo (SP) – Brazil, 2013 to 2019.

During the study period, 481 older adults with femoral fractures were admitted to the institution and, of these, 295 (61.3%) were admitted to the ICU. As for the historical series, Figure 1 shows that from 2017 onwards there was a significant increase in hospitalizations of older adults with femoral fractures in the ICU, reaching a peak in 2019 (66 cases). Furthermore, the red line in Figure 1 shows that, despite no relationship being observed between age and monthly number of admissions, the average age of patients was over 75 years old in almost all of the months analyzed.

Data in Table 1 show the characterization of the sample regarding older adults with femoral fractures admitted to the ICU. There was a higher frequency of female patients (72.2%) and the majority (55.6%) were aged 80 years or over. Charlson comorbidity index ranged from 1 to 8 points and 288 patients (97.6%) obtained a score greater than or equal to 3. Falls were the most common cause of femur fractures (98.6%), especially from own height (88.1%). Among the 260 patients who suffered this type of fall, 69.2% were over 80 years old. The region of femur fracture with the highest incidence was the distal (53.6%) and surgical treatment prevailed in the sample (92.3%).

**Table 1:** Characterization of sample patients. São Paulo (SP) – Brazil, 2013 to 2019.

Variables	n (%)	Mean (SD)	Median (1st – 3rd quartile)
Sex			
Female	213 (72.2)		
Male	82 (27.8)		
Age		82.3 (8.3)	83 (78 – 101)
Charlson comorbidity index		4.4 (1,1)	4 (4 – 5)
Cause of femur fracture			
Fall from own height	260 (88.1)		
Fall from one level to another	31 (10.5)		
Others*	4 (1.4)		
Anatomical region of the fracture			
Diaphysis	23 (7.8)		
Distal	158 (53.6)		
Proximal	114 (38.6)		
Type of treatment			
Surgical	284 (92.3)		
Non-surgical	11 (3.7)		
Risk of death according to SAPS 3		14.6 (15.9)	8.4 (4.7 – 17.1)
NAS ICU admission		67.7 (17.3)	66.1 (53.7 – 79.7)
NAS stay in the ICU		59.3 (13.1)	56.4 (50.8 – 66.3)
Length of stay in the ICU (in days)		4.7 (5.7)	2.9 (1.1 – 6.0)
Clinical outcome in the ICU			
Survivor	241 (81.7)		
Non-survivor	54 (18.3)		

* Pathological fracture (n = 2), being run over (n = 1), crushing (n = 1).

The risk of death according to SAPS 3 ranged from 0.4% to 90.3%, with 13 patients having a risk of dying greater than 50% and, among them, 7 (53.8%) died. The mean and median NAS upon admission of patients to the ICU exceeded the values calculated during their length of stay in the unit. Approximately 42.0% of patients were hospitalized in the critical unit for four days or more. Regarding the clinical outcome in the ICU, 54 older people (18.3%) died and all patients undergoing conservative treatment (n = 11) died during intensive treatment.

SD: Standard deviation; SAPS 3: Simplified Acute Physiology Score version 3; NAS: Nursing Activities Score; ICU: Intensive care unit

In Table 2, it can be observed that there was a significant difference between age and the variables sex (p = 0.007), anatomical region of the femur fracture (p = 0.047), and clinical outcome in the ICU (p = 0.015). Female patients with distal femur fractures who died in the ICU had higher median age than men, patients with shaft or proximal femur fractures, and survivors.

**Table 2:** Association between age and sex, characteristics and treatment of femoral fracture and clinical evolution of patients in the ICU. São Paulo (SP) – Brazil, 2013 to 2019.

Variables	Age	p
	Median (1st – 3rd quartile)	
Sex		
Female	84 (79 – 88)	0.007*
Male	81 (74 – 86.7)	
Cause of femur fracture		
Fall from own height	83 (78 – 88)	0.176**
Fall from one level to another	83 (76.5 – 87.5)	
Other	78.5 (74.5 – 80.2)	
Anatomical region of the fracture		
Diaphysis	80 (76 – 85.5)	0.047**
Distal	84 (79 – 88)	
Proximal	82.5 (76.2 – 88)	
Type of treatment		
Surgical	83 (78 – 88)	0.616*
Non-surgical	85 (79.5 – 88.5)	
Clinical outcome in the ICU		
Survivor	83 (77 – 87)	0.015*
Non-survivor	85.5 (82 – 89.7)	

* Wilcoxon-Mann-Whitney Test;

** Kruskal-Wallis Test ICU: Intensive care unit.

**Figure 2:**
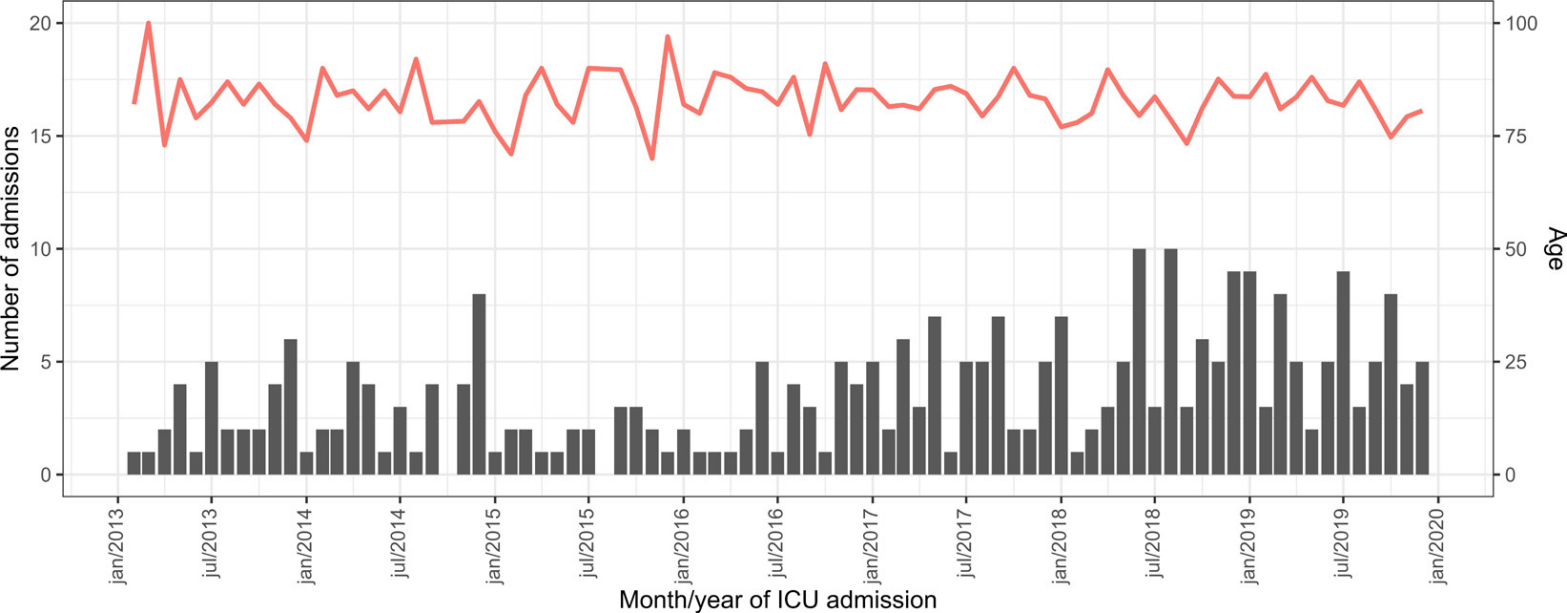
Scatterplots between age and variables related to comorbidity, nursing workload, severity, and length of stay in the ICU. São Paulo (SP) – Brazil, 2013 to 2019.

Data in Table 3 and Figure 2 show that there was a weak and positive correlation between age and Charlson comorbidity index (r = 0.354) and no correlation with the other variables (r values lower than 0.10).

**Table 3:** Correlation between age and variables related to comorbidity, nursing workload, severity, and length of stay in the ICU. São Paulo (SP) – Brazil, 2013 to 2019.

Variables	r*	95%CI	
		Min	Max
Charlson comorbidity index	0.354	0.250	0.450
NAS ICU admission	–0.059	–0.173	0.055
NAS stay in the ICU	–0.003	–0.117	0.111
Risk of death according to SAPS 3	0.060	–0.055	0.173
Length of stay in the ICU (in days)	0.029	–0.085	0.143

* Pearson Correlation Coefficient; CI: Confidence Interval; Min Minimum: Max: Maximum; NAS: Nursing Activities Score; ICU: Intensive care unit Simplified Acute Physiology Score version 3.

## DISCUSSION

The study results showed a significant increase in ICU admissions of older adults with femoral fractures and characteristics related to the patient (sex), the injury (anatomical region of the fracture), and the clinical outcome in the ICU that were associated with age.

Researchers point out that osteosarcopenia increases the risk of falls and the occurrence of fractures in older individuals^(18)^. Furthermore, it is observed that older people over 80 years of age are more vulnerable to frailty and, consequently, to the occurrence of fractures due to factors related to unintentional weight loss, reduced physical activity level, slow walking speed, low grip strength, and self-reported exhaustion^(19,20)^. These conditions lead to an increased incidence of fractures after a fall in the older adults, the need for hospital admission and, in cases of vulnerability and associated comorbidities, admission to the ICU.

Falls from own height, especially among older women, were the most frequently found in a population-based study that analyzed the characteristics of this type of trauma^(21)^, corroborating the findings of this investigation. Regarding the location of the injury, a Danish study observed a general incidence of fractures of the distal femur of 8.7 per 100,000 inhabitants per year, with an exponential increase in this type of fracture, in both sexes, after the age of 60^(22)^. Therefore, it is extremely important to identify patients at high risk of dying after a femur fracture^(5)^. This information can help professionals in a careful clinical assessment at the bedside and in the development of a systematized and assertive care plan for hospitalized older people, aiming at reducing complications and deaths.

As for the NAS average, admission values exceeded those for ICU stay, similar to the findings of the study that explored the nursing workload required by clinical and surgical patients admitted to the ICU of a hospital in Florence, Italy, and found that interventions related to admission procedures in the critical care unit are items that most interfered with the demand for care^(23)^. Regarding the length of stay in the ICU, it is worth highlighting that older people with some frailty criteria, such as partially or totally dependent functional health status, diabetes mellitus, history of chronic obstructive pulmonary disease, congestive heart failure or systemic arterial hypertension that requires medication^(24)^, have a 20 times greater risk of remaining longer than 6 days after a femur fracture^(25)^.

In the analysis of the variables that were associated with age, it was found that women and patients with distal femur fractures had a higher median age. A study analyzing data from the hospital information system of the Brazilian Public Health System showed a higher incidence (1.7 times) of hospitalizations of women due to femoral fractures and high lethality in this population^(26)^. From the age of 60 onwards, females are responsible for the highest number of hospitalizations for treatment of femoral fractures, with emphasis on the significant increase in cases from the age of 70 onwards^(27)^. Risk factors such as reduced bone mineral density and hip joint load, in addition to sarcopenia, are associated with femur fractures in the older population, especially in women^(4,28,29)^.

Although the present study did not find any correlation between age and SAPS 3, the risk of death estimated by the index (14.6%) was close to that observed in the sample (18.3%). For the 11 older adults undergoing conservative treatment, the mortality rate during ICU length of stay was 100%. In this regard, the severity of these older adults may justify the higher mortality observed because, according to medical records, these patients were not clinically fit for surgical treatment. Non-surgical treatment, inability to walk before the fracture, and age over 80 years are conditions that increase the risk of death^(5)^, which corroborates the findings of the present investigation, since 9 of the 11 older patients who did not undergo surgery to treat the injury were over 80 years of age.

The data from this study showed a significant number of older adults (61.3%) who had a femur fracture and required admission to the ICU. In this regard, the importance of improving fall prevention policies, ensuring a healthy life, and promoting the older population well-being as set out in the Sustainable Development Goals for Health and Well-being (SDG 3) for the United Nations (UN) 2030 agenda^(30)^ is highlighted. Moreover, this study was pioneering in analyzing the nursing workload measured by the NAS in the older population with femoral fractures, thus bringing important contributions that can help in the dimensioning of the nursing team and the quality of care provided to patients in the ICU.

Some limitations of this research must be considered. The type of study, characterized by the retrospective analysis of the older adults’ medical records, directly depends on the quality and completeness of the records made by the professionals. Furthermore, the research was carried out in just one service, of secondary and medium complexity care, and this fact must be considered in the external generalization of the findings. It is also worth mentioning that the institution of the present study was referenced, from 2017, for the care of patients with orthopedic diseases who required immediate post-operative care in the ICU, which may justify the significant increase in admissions from this year onwards.

Finally, the results of this study allowed us to understand the characteristics of older adults who require intensive care due to femoral fractures and bring important findings that can support ICU professionals, especially nurses, in the development of a care plan and management of human resources that meet the needs of this population and ensure safe assistance. In addition, the data expressed in this research show the need to strengthen public policies aimed at preventing falls, including the recognition of factors contributing to the event, encouragement of the practice of physical activity, implementation of personalized interventions for vulnerable older people and with a high risk of falls, and family guidance, thus reducing the occurrence of this type of injury and the need for admission to the ICU.

## CONCLUSION

The study showed, through the analysis of the historical series, an increase in hospitalizations of older adults with femoral fractures in the ICU from 2017 onwards. The results also allowed us to conclude that female patients, with distal femur fractures and who died in the ICU, were older than men, patients with shaft or proximal femur fractures, and survivors.

The study emphasizes the importance of ICU health professionals understanding the characteristics and needs of older people with femoral fractures to structure intensive care, implement care protocols, and train the team to care for this very specific population. In addition, further studies should be carried out in other Brazilian services and states to expand knowledge on this population and achieve future benchmarking to improve the quality of care and, mainly, the strategies for prevention of falls, which are still so common in our population.

## References

[1] Instituto Brasileiro de Geografia e Estatística (2023). Censo Brasileiro de 2022.

[2] Yeung SSY, Reijnierse EM, Pham VK, Trappenburg MC, Lim WK, Meskers CGM (2019). Sarcopenia and its association with falls and fractures in older adults: a systematic review and meta-analysis. J Cachexia Sarcopenia Muscle.

[3] Cuevas-Trisan R (2019). Balance problems and fall risks in the elderly. Clin Geriatr Med.

[4] Liu P, Zhang Y, Sun B, Chen H, Dai J, Yan L (20218). Risk factors for femoral neck fracture in elderly population. Zhong Nan Da Xue Xue Bao Yi Xue Ban.

[5] Merino-Rueda LR, Rubio-Sáez I, Mills S, Rubio-Suárez JC (2021). Mortality after distal femur fractures in the elderly. Injury.

[6] Ibrahim YB, Mohamed AY, Ibrahim HS, Mohamed AH, Cici H, Mohamed YG (2023). Risk factors, classification, and operative choices of femur fractures at a Tertiary Hospital: first report from Somalia. Sci Rep.

[7] Merloz P (2018). Optimization of perioperative management of proximal femoral fracture in the elderly. Orthop Traumatol Surg Res.

[8] Moreira RS, Souza JG, Siqueira AR, Xavier MD, Oliveira SP, Bauman CD (2021). Mortalidade em idosos com fratura de fêmur proximal em um hospital universitário. REAS/EJCH.

[9] Peterle VCU, Geber JC, Darwin W, Lima AV, Bezerra PE, Novaes MRCG (2020). Indicators of morbidity and mortality by femur fractures in older people: a decade-long study in brazilian hospitals. Acta Ortop Bras.

[10] Maffulli N, Aicale R (2022). Proximal femoral fractures in the elderly: a few things to know, and some to forget. Medicina (Kaunas).

[11] Li Q, Wang Y, Shen X (2022). Improvement of negative psychological stress response in elderly patients with femoral neck fracture by integrated high-quality nursing model of medical care. Front Surg.

[12] Ferretti-Rebustini REL, Nogueira LS, Silva RCG, Poveda VB, Machado SP, Oliveira EM (2017). Aging as a predictor of nursing workload in Intensive Care Unit: results from a Brazilian Sample. Rev Esc Enferm USP.

[13] Association for the Advancement of Automotive Medicine (2015). The Abbreviated Injury Scale (AIS): 2008, update 2015.

[14] Charlson ME, Pompei P, Ales KL, MacKenzie CR (1987). A new method of classifying prognostic comorbidity in longitudinal studies: development and validation. J Chronic Dis.

[15] Moreno RP, Metnitz PG, Almeida E, Jordan B, Bauer P, Campos RA (2005). SAPS 3 – From evaluation of the patient to evaluation of the intensive care unit. Part 2: development of a prognostic model for hospital mortality at ICU admission. Intensive Care Med.

[16] Miranda DR, Nap R, de Rijk A, Schaufeli W, Iapichino G (2003). Nursing Activities Score. Crit Care Med.

[17] Queijo AF, Padilha KG (2009). Nursing Activities Score (NAS): cross-cultural adaptation and validation to Portuguese language. Rev Esc Enferm USP.

[18] Kirk B, Zanker J, Duque G (2020). Osteosarcopenia: epidemiology, diagnosis, and treatment-facts and numbers. J Cachexia Sarcopenia Muscle.

[19] Hoogendijk EO, Afilalo J, Ensrud KE, Kowal P, Onder G, Fried LP (2019). Frailty: implications for clinical practice and public health. Lancet.

[20] Fischer H, Maleitzke T, Eder C, Ahmad S, Stöckle U, Braun KF (2021). Management of proximal femur fractures in the elderly: current concepts and treatment options. Eur J Med Res.

[21] Moraes SA, Soares WJS, Lustosa LP, Bilton TL, Ferrioli E, Perracini MR (2017). Characteristics of falls in elderly persons residing in the community: a population-based study. Rev Bras Geriatr Gerontol.

[22] Elsoe R, Ceccotti AA, Larsen P (2018). Population-based epidemiology and incidence of distal femur fractures. Int Orthop.

[23] Forciniti C, Lucchini A, Pietrini L, Rasero L, Bambi S (2022). Measuring the nursing workload in a medical-surgical high dependency unit through nursing activities score (NAS). A prospective observational study. Assist Inferm Ric.

[24] Subramaniam S, Aalberg JJ, Soriano RP, Divino CM (2021). The 5-factor modified frailty index in the geriatric surgical population. Am Surg.

[25] Aizpuru M, Staley C, Reisman W, Gottschalk MB, Schenker ML (2018). Determinants of length of stay after operative treatment for femur fractures. J Orthop Trauma.

[26] Vasconcelos PAB, Rocha AJ, Fonseca RJS, Teixeira TRG, Mattos ESR, Guedes A (2020). Femoral fractures in the elderly in Brasil – incidence, lethality, and costs (2008–2018). Rev Assoc Med Bras.

[27] Andrade JP, Silva DZ, Patrício DS (2020). Incidência dos casos de fratura de fêmur no Brasil entre 2015 e 2020 através de dados epidemiológicos do DATASUS: faixa etária e gênero. Sci Gen.

[28] Tsuda T (2017). Epidemiology of fragility fractures and fall prevention in the elderly: a systematic review of the literature. Curr Orthop Pract.

[29] Larsson L, Degens H, Li M, Salviati L, Lee YI, Thompson W (2019). Sarcopenia: aging-related loss of muscle mass and function. Physiol Rev.

[30] Cruz DKA, Nóbrega AA, Montenegro MMS, Pereira VOM (2022). The Sustainable Development Goals and data sources for monitoring goals in Brazil. Epidemiol Serv Saude.

