# Supplementary material for: Analysis of intensive care unit admissions for older adults with femoral fractures: a retrospective cohort
Source: Rev Esc Enferm USP. 2024 Aug 2;58:e20230398. doi: 10.1590/1980-220X-REEUSP-2023-0398en (PMC11299534; doi:10.1590/1980-220X-REEUSP-2023-0398en)
Supplement: Translation

# Translation: Análise das internações na unidade de terapia intensiva de idosos com fratura de fêmur: coorte retrospectivo

**DOI:** 10.1590/1980-220X-REEUSP-2023-0398pt

**Authors:** Carolina Rodrigues Mendes Nogueira Cobra, Paulo Carlos Garcia, Isadora Castilho Moreira de Oliveira Passos, Greiciane da Silva Rocha, Lilia de Souza Nogueira

**Affiliations:** 1 Universidade de São Paulo, Escola de Enfermagem, São Paulo, SP, Brasil.; 2 Universidade de São Paulo, Hospital Universitário, São Paulo, SP, Brasil.; 3 Universidade Federal do Acre, Centro de Ciências da Saúde e do Desporto, Rio Branco, AC, Brasil.

**Keywords:** Fraturas do Fêmur, Idoso, Unidades de Terapia Intensiva, Carga de Trabalho, Mortalidade

## Abstract

**Objetivos:**

Descrever a série histórica das internações, na Unidade de Terapia Intensiva, de idosos com fratura de fêmur, e verificar a associação entre idade e as características e tratamento da lesão, carga de trabalho de enfermagem, gravidade e evolução clínica na unidade.

**Método:**

Coorte retrospectiva de 295 idosos (idade ≥60 anos) admitidos na Unidade de Terapia Intensiva de hospital em São Paulo, entre 2013 e 2019, e que apresentaram fratura de fêmur como causa principal da internação. Variáveis sobre características demográficas, causa e tipo da fratura, tratamento realizado, gravidade, carga de trabalho de enfermagem e desfecho clínico dos pacientes foram analisadas. Os testes de *Shapiro-Wilk*, *Wilcoxon-Mann-Whitney*, *Kruskal-Wallis* e correlação de Pearson foram aplicados.

**Resultados:**

Houve aumento das internações de idosos na Unidade de Terapia Intensiva a partir de 2017. Pacientes do sexo feminino, com fratura distal de fêmur e que morreram na Unidade de Terapia Intensiva apresentaram significativamente (p < 0,05) maior mediana de idade do que homens, pacientes com fratura de diáfise ou proximal do fêmur e sobreviventes.

**Conclusão:**

Os achados do estudo evidenciam informações essenciais para estruturação dos cuidados ao idoso com fratura de fêmur que necessita de tratamento intensivo.

## INTRODUÇÃO

Desde a década de 70, observa-se transição sociodemográfica no Brasil com aumento expressivo da proporção de idosos em comparação a qualquer outra faixa etária. Segundo o Instituto Brasileiro de Geografia e Estatística (IBGE), o país apresentará crescimento populacional significativo de idosos até 2039 e, ainda, em 2050, este grupo ultrapassará o das crianças, transformando o Brasil em um dos países mais envelhecidos do mundo^(1)^.

O processo do envelhecimento é caracterizado, fisiologicamente, por redução da massa muscular e perda orgânica e funcional que interferem diretamente na maior dependência e necessidade de cuidados, além da alteração no equilíbrio e déficit na marcha, que podem aumentar o risco de queda e, consequentemente, a ocorrência de fratura de fêmur em idosos^(2,3)^. Estudo mostra que, além da idade avançada, variáveis relacionadas ao sexo feminino, baixo índice de massa corporal, presença de osteoporose, maior comprimento e menor circunferência média da coxa são fatores de risco para a ocorrência de fratura de fêmur na população idosa^(4)^.

As fraturas de fêmur podem ocorrer na região proximal, distal ou na diáfise femoral^(5)^. A fratura de fêmur na região proximal é a mais frequente e decorrente, principalmente, da queda da própria altura de pessoas com mais de 60 anos^(6)^. O impacto da fratura de fêmur nas condições de saúde do idoso é notório, pois este tipo de lesão contribui para o aumento da fragilidade e da dependência, reduzindo a qualidade de vida^(7,8)^. Em relação ao desfecho clínico, estudo de série temporal mostrou que, entre 2008 e 2018, a taxa média de mortalidade de idosos em decorrência de fratura de fêmur foi de 5%, sendo mais elevada entre aqueles com idade superior a 80 anos^(9)^.

Portanto, este tipo de fratura, prevalente em idosos, acarreta hospitalização e tratamento cirúrgico e representa elevado custo social e econômico em decorrência do prolongado tempo de permanência hospitalar e de períodos de reabilitação^(9,10)^. Ademais, a ocorrência de complicações no pós-operatório da correção da fratura de fêmur, muitas vezes associada à presença de comorbidades e à vulnerabilidade dos idosos, remete à necessidade de vigilância contínua na Unidade de Terapia Intensiva (UTI) com foco na redução de complicações e mortes^(7,8,10)^.

Neste sentido, a atuação do enfermeiro junto à equipe multidisciplinar no cuidado ao idoso com fratura de fêmur internado na UTI é essencial e inclui ações de prevenção de complicações, como infecções relacionadas à assistência à saúde (IRAS) e lesão por pressão (LPP), controle da dor, cuidados psicológicos, reabilitação o mais precocemente possível, entre outras^(11)^. Ainda sobre cuidados ao paciente na UTI, pesquisadores identificaram que a carga de trabalho de enfermagem requerida por idosos durante a permanência na unidade crítica é mais elevada em comparação aos adultos^(12)^, reforçando assim a importância da análise da demanda de cuidados desta população para adequação quantitativa e qualitativa de recursos humanos com foco na garantia da qualidade assistencial.

Apesar de o estudo citado anteriormente^(12)^ destacar a elevada carga de trabalho de enfermagem de idosos na UTI, a amostra analisada foi composta por pacientes com diferentes patologias, o que pode não refletir as características de uma população tão específica, como a de idosos com fratura de fêmur que necessitam de cuidados intensivos. Além disso, há lacuna de conhecimento na literatura sobre a possível associação entre a idade dessa população e as variáveis relacionadas às características da lesão, tratamento realizado para correção da fratura de fêmur, gravidade e desfecho clínico na UTI.

Diante disso, justifica-se a realização do estudo em tela cujos resultados poderão contribuir, na prática clínica, para o reconhecimento das possíveis características e necessidades geriátricas que podem auxiliar na promoção de um melhor processo de enfermagem (PE) voltado ao público idoso, de modo a enfocar em diagnósticos, resultados e intervenções prioritárias, na capacitação dos profissionais para assistência ao idoso com fratura de fêmur internado na UTI e na melhor gestão de recursos humanos e da qualidade do cuidado prestado, a fim de minimizar complicações e reduzir o desgaste profissional. Nesse sentido, o presente estudo tem como objetivos descrever a série histórica das internações na UTI de idosos com fratura de fêmur e verificar a associação entre idade e as características e tratamento da lesão, carga de trabalho de enfermagem, gravidade e evolução clínica na unidade.

## MÉTODO

### Desenho do Estudo

Trata-se de um estudo de coorte retrospectivo que analisou prontuários de pacientes idosos (≥ 60 anos) internados na UTI.

### Local

O estudo foi realizado em um hospital universitário localizado na cidade de São Paulo, Brasil. A instituição possui uma UTI geral, com 12 leitos, que presta assistência a pacientes críticos clínicos e cirúrgicos.

### População e Critérios de Seleção

A casuística foi composta por idosos admitidos na UTI entre 1º de janeiro de 2013 e 31 de dezembro de 2019, e que apresentaram fratura de fêmur como causa da internação hospitalar e ferimento principal, caracterizado pela ausência de lesão, de acordo com a *Abbreviated Injury Scale (AIS)* ^(13)^, maior ou igual a 3 em outra região corpórea.

A AIS é uma escala utilizada para descrever as lesões decorrentes do trauma e identificar sua gravidade, classificando-as em: AIS 1 (lesão leve), AIS 2 (lesão moderada), AIS 3 (lesão grave), AIS 4 (lesão grave com risco iminente de vida), AIS 5 (lesão crítica, de sobrevida incerta) ou AIS 6 (lesão de gravidade máxima, incompatível com a vida)^(13)^. A fratura de fêmur é classificada como lesão AIS 3, segundo o manual AIS^(13)^.

### Variáveis

As variáveis analisadas no estudo foram idade, sexo, causa da fratura de fêmur (queda da própria altura, queda de um nível a outro ou outras causas), região anatômica da fratura (proximal, distal ou diáfise de fêmur), tipo de tratamento (cirúrgico ou conservador), tempo de permanência (em dias) e desfecho clínico na UTI (sobrevivente ou não sobrevivente), índice de comorbidade de *Charlson* ^(14)^, risco de morte segundo o *Simplified Acute Physiology Score* versão 3 (SAPS 3)^(15)^ e carga de trabalho de enfermagem mensurada pelo *Nursing Activities Score* (NAS)^(16)^.

O índice de comorbidade de *Charlson* é um método prognóstico que avalia 19 condições clínicas prévias do paciente e, para cada uma delas, atribui pontuação de zero a seis. Esses pontos são somados e o valor final do índice reflete o peso das comorbidades do paciente na mortalidade, ou seja, maiores escores do índice estão associados com aumento da frequência de mortes^(14)^. O SAPS 3 é um escore prognóstico composto por 20 variáveis subdivididas em demográficas, motivos da admissão na UTI e fisiológicas, que representam o grau de comprometimento da doença e permitem definir, por meio de uma equação de regressão logística, o risco de morte do paciente na UTI^(15)^.

O NAS é um instrumento de mensuração da carga de trabalho de enfermagem composto por 23 atividades de enfermagem distribuídas em sete grandes categorias (atividades básicas, suportes ventilatório, cardiovascular, renal, neurológico e metabólico e intervenções específicas), sendo que cada atividade realizada atribui um peso no escore final. O NAS permite identificar a porcentagem de tempo gasto pelo profissional de enfermagem, na assistência direta e indireta ao paciente, nas últimas 24 horas de internação na UTI, podendo atingir o máximo de 176,8^(16)^. Pesquisadores realizaram a adaptação transcultural e validação do NAS para a língua portuguesa e identificaram que o instrumento apresentou índices satisfatórios de confiabilidade, de validade de critério e de constructo, sendo considerado um indicador válido e confiável para mensurar a carga de trabalho de enfermagem em UTI do Brasil^(17)^.

### Coleta de Dados

O período de análise das admissões iniciou-se em 2013, ano da informatização do instrumento de medida de carga de trabalho de enfermagem na UTI do estudo e foi finalizado em dezembro de 2019, pois, em 2020, houve alteração significativa do perfil de admissões na UTI devido à pandemia da COVID-19.

Para a coleta dos dados, foram aplicados quatro instrumentos: dados demográficos, relacionados à fratura do fêmur e evolução clínica na UTI (instrumento 1), cálculo do índice de comorbidade de *Charlson* (instrumento 2), escore NAS (instrumento 3) e cálculo do SAPS 3 (instrumento 4). As informações foram coletadas dos prontuários físicos dos pacientes e do sistema informatizado da UTI. Para o índice SAPS 3, foram considerados os dados da primeira hora de internação do paciente na UTI e o cálculo foi realizado na planilha disponível em site de domínio público do SAPS3 *Research Group* (https://www.saps3.org/archive/downloads/user-agreement/downloads/). As informações do NAS admissão (referente às primeiras 24 horas do paciente na unidade) e NAS permanência na UTI (referente aos valores do NAS durante toda a permanência na unidade crítica) foram resgatadas do sistema informatizado da UTI que é alimentado diariamente pelos enfermeiros na unidade, treinados periodicamente para o cálculo do escore.

### Análise e Tratamento dos Dados

Os dados foram tabulados no programa *Microsoft Excel* ^®^ 2013 e analisados com o apoio de um profissional estatístico por meio do programa R versão 4.1.2. Para análise da série histórica proposta no objetivo do estudo, foi construído um gráfico combinado de barras e linha descrevendo o número de admissões mensais na UTI de idosos com fratura de fêmur e a idade média dos pacientes. Para verificar a associação entre idade e as variáveis categóricas do estudo, primeiramente foi aplicado o teste *Shapiro-Wilk* que identificou distribuição não normal da idade dos pacientes na amostra. Portanto, os testes não paramétricos *Wilcoxon-Mann-Whitney* e *Kruskal-Wallis* foram utilizados nas análises de associação. Em relação à correlação entre idade e as variáveis numéricas, foi calculado o coeficiente de correlação de *Pearson*, sendo considerada nenhuma correlação o valor do coeficiente de 0,00 a 0,10; fraca correlação, de 0,10 a 0,39; moderada correlação, de 0,40 a 0,69; forte correlação, de 0,70 a 0,89; e muito forte correlação, de 0,90 a 1,00. Em todas as análises, foi estabelecido o nível de significância de 5%.

### Aspectos Éticos

A pesquisa foi aprovada em 2021 pelo Comitê de Ética em Pesquisa (CEP) da instituição (Parecer nº 4.789.403) e, por se tratar de análise retrospectiva de prontuários dos pacientes, o CEP dispensou a aplicação do Termo de Consentimento Livre e Esclarecido aos participantes.

## RESULTADOS

No período do estudo, foram admitidos na instituição 481 idosos com fratura de fêmur e, destes, 295 (61,3%) foram internados na UTI. Quanto à série histórica, a Figura 1 evidencia que a partir de 2017 houve um aumento importante de internações de idosos com fratura de fêmur na UTI, atingindo o pico em 2019 (66 casos). Ademais, a linha vermelha da Figura 1 mostra que, apesar de não se observar relação entre idade e número mensal de admissões, a idade média dos pacientes superou os 75 anos na quase totalidade dos meses analisados.

**Figura 1:** Distribuição mensal das admissões na UTI de idosos com fratura de fêmur e idade média dos pacientes. São Paulo (SP) – Brasil, 2013 a 2019.

Os dados da Tabela 1 mostram a caracterização da amostra referente aos idosos com fratura de fêmur internados na UTI. Observa-se maior frequência de pacientes do sexo feminino (72,2%) e a maioria (55,6%) tinha idade igual ou superior a 80 anos. O índice de comorbidade de *Charlson* variou de 1 a 8 pontos e 288 pacientes (97,6%) obtiveram escore maior ou igual a 3. As quedas foram a causa mais frequente da fratura de fêmur (98,6%), especialmente da própria altura (88,1%). Entre os 260 pacientes que sofreram este tipo de queda, 69,2% tinham idade superior a 80 anos. A região da fratura de fêmur de maior incidência foi a distal (53,6%) e o tratamento cirúrgico prevaleceu na amostra (92,3%).

**Tabela 1:** Caracterização dos pacientes da amostra. São Paulo (SP) – Brasil, 2013 a 2019.

Variáveis	n (%)	Média (DP)	Mediana (1º – 3º quartil)
Sexo			
Feminino	213 (72,2)		
Masculino	82 (27,8)		
Idade		82,3 (8,3)	83 (78 – 101)
Índice de comorbidade de *Charlson*		4,4 (1,1)	4 (4 – 5)
Causa da fratura de fêmur			
Queda da própria altura	260 (88,1)		
Queda de um nível a outro	31 (10,5)		
Outras*	4 (1,4)		
Região anatômica da fratura			
Diáfise	23 (7,8)		
Distal	158 (53,6)		
Proximal	114 (38,6)		
Tipo de tratamento			
Cirúrgico	284 (92,3)		
Conservador	11 (3,7)		
Risco de morte segundo SAPS 3		14,6 (15,9)	8,4 (4,7 – 17,1)
NAS admissão na UTI		67,7 (17,3)	66,1 (53,7 – 79,7)
NAS permanência na UTI		59,3 (13,1)	56,4 (50,8 – 66,3)
Tempo de permanência na UTI (em dias)		4,7 (5,7)	2,9 (1,1 – 6,0)
Desfecho clínico na UTI			
Sobrevivente	241 (81,7)		
Não sobrevivente	54 (18,3)		

* Fratura patológica (n = 2), atropelamento (n = 1), esmagamento (n = 1); DP: Desvio-Padrão; SAPS 3: *Simplified Acute Physiology Score* versão 3; NAS: *Nursing Activities Score*; UTI: Unidade de Terapia Intensiva.

O risco de morte segundo SAPS 3 variou de 0,4% a 90,3%, sendo que 13 pacientes apresentaram risco de morrer superior a 50% e, entre eles, 7 (53,8%) foram a óbito. A média e a mediana do NAS na admissão dos pacientes na UTI superaram os valores calculados durante a permanência na unidade. Aproximadamente 42,0% dos pacientes ficaram internados na unidade crítica por quatro dias ou mais. Em relação ao desfecho clínico na UTI, 54 idosos (18,3%) foram a óbito e todos os pacientes submetidos a tratamento conservador (n = 11) morreram durante o tratamento intensivo.

Observa-se na Tabela 2 que houve diferença significativa entre a idade e as variáveis sexo (p = 0,007), região anatômica da fratura de fêmur (p = 0,047) e desfecho clínico na UTI (p = 0,015). Os pacientes do sexo feminino, com fratura distal de fêmur e que morreram na UTI apresentaram maior mediana de idade do que homens, pacientes com fraturas de diáfise ou proximal do fêmur e sobreviventes.

**Tabela 2:** Associação entre idade e sexo, características e tratamento da fratura de fêmur e evolução clínica dos pacientes na UTI. São Paulo (SP) – Brasil, 2013 a 2019.

Variáveis	Idade	p
	Mediana (1º – 3º quartil)	
Sexo		
Feminino	84 (79 – 88)	0,007*
Masculino	81 (74 – 86,7)	
Causa da fratura de fêmur		
Queda da própria altura	83 (78 – 88)	0,176**
Queda de um nível a outro	83 (76,5 – 87,5)	
Outras	78,5 (74,5 – 80,2)	
Região anatômica da fratura		
Diáfise	80 (76 – 85,5)	0,047**
Distal	84 (79 – 88)	
Proximal	82,5 (76,2 – 88)	
Tipo de tratamento		
Cirúrgico	83 (78 – 88)	0,616*
Conservador	85 (79,5 – 88,5)	
Desfecho clínico na UTI		
Sobrevivente	83 (77 – 87)	0,015*
Não sobrevivente	85,5 (82 – 89,7)	

* Teste *Wilcoxon-Mann-Whitney*;

** Teste *Kruskal-Wallis;* UTI: Unidade de Terapia Intensiva.

Os dados da Tabela 3 e Figura 2 mostram que houve correlação fraca e positiva entre a idade e o índice de comorbidade de *Charlson* (r = 0,354) e nenhuma correlação com as outras variáveis (valores de r inferiores a 0,10).

**Tabela 3:** Correlação entre idade e as variáveis relacionadas à comorbidade, carga de trabalho de enfermagem, gravidade e tempo de permanência na UTI. São Paulo (SP) – Brasil, 2013 a 2019.

Variáveis	r*	IC95%	
		Mín	Máx
Índice de comorbidade de *Charlson*	0,354	0,250	0,450
NAS admissão na UTI	–0,059	–0,173	0,055
NAS permanência na UTI	–0,003	–0,117	0,111
Risco de morte segundo SAPS 3	0,060	–0,055	0,173
Tempo de permanência na UTI (em dias)	0,029	–0,085	0,143

* Coeficiente de Correlação de *Pearson;* IC: Intervalo de Confiança; Mín: Mínimo: Máx: Máximo; NAS: *Nursing Activities Score*; UTI: Unidade de Terapia Intensiva; SAPS 3: *Simplified Acute Physiology Score* versão 3.

**Figura 2:** Gráficos de dispersão entre idade e as variáveis relacionadas à comorbidade, carga de trabalho de enfermagem, gravidade e tempo de permanência na UTI. São Paulo (SP) – Brasil, 2013 a 2019.

## DISCUSSÃO

Os resultados do estudo evidenciaram aumento significativo das internações na UTI de idosos com fratura de fêmur e características relacionadas ao paciente (sexo), à lesão (região anatômica da fratura) e ao desfecho clínico na UTI que se associaram com a idade.

Pesquisadores apontam que a osteosarcopenia aumenta o risco de queda e a ocorrência de fraturas na população idosa^(18)^. Ademais, observa-se que idosos acima de 80 anos apresentam maior vulnerabilidade à fragilidade e, consequentemente, à ocorrência de fraturas devido a fatores relacionados à perda de peso não intencional, reduzido nível de atividade física, velocidade de caminhada lenta, baixa força de preensão e autorrelato de exaustão^(19,20)^. Essas condições acarretam aumento da incidência de fratura após queda em idosos, necessidade de internação hospitalar e, em casos de vulnerabilidade e comorbidades associadas, internação na UTI.

Quedas da própria altura, especialmente de mulheres idosas, foram as mais frequentemente encontradas em estudo de base populacional que analisou as características deste tipo de trauma^(21)^, corroborando os achados desta investigação. Quanto à localização da lesão, estudo dinamarquês identificou incidência geral de fraturas do fêmur distal de 8,7 por 100.000 mil habitantes ao ano, com aumento exponencial deste tipo de fratura, em ambos os sexos, após os 60 anos^(22)^. Diante disso, é de extrema relevância identificar os pacientes com alto risco de morrer após a fratura do fêmur^(5)^. Essas informações podem auxiliar os profissionais na avaliação clínica criteriosa a beira leito e na elaboração de um plano de cuidados sistematizado e assertivo ao idoso hospitalizado com o objetivo de reduzir complicações e mortes.

Quanto à média do NAS, os valores de admissão superaram os da permanência na UTI, semelhante ao achado do estudo que explorou a carga de trabalho de enfermagem requerida por pacientes clínicos e cirúrgicos internados na UTI de um hospital em Florença, Itália, e identificou que intervenções relacionadas a procedimentos admissionais na unidade crítica são itens que mais interferiram na demanda de cuidados^(23)^. No tocante ao tempo de permanência na UTI, vale destacar que idosos com algum critério de fragilidade, como estado de saúde funcional parcialmente ou totalmente dependente, diabetes mellitus, histórico de doença pulmonar obstrutiva crônica, insuficiência cardíaca congestiva ou hipertensão arterial sistêmica que requer medicação^(24)^, têm risco 20 vezes maior de permanecer tempo superior a 6 dias após fratura de fêmur^(25)^.

Na análise das variáveis que se associaram à idade, verificou-se que as mulheres e pacientes com fratura distal de fêmur apresentaram maior mediana de idade. Estudo que analisou dados do sistema de informações hospitalares do Sistema Único de Saúde mostrou maior incidência (1,7 vezes) de internações de mulheres por fratura de fêmur e elevada letalidade nesta população^(26)^. A partir dos 60 anos, o sexo feminino é responsável pelo maior número de internações para tratamento de fratura de fêmur, com destaque ao aumento expressivo de casos a partir dos 70 anos^(27)^. Fatores de risco como redução da densidade mineral óssea e carga das articulações do quadril, além da sarcopenia, estão associados à fratura de fêmur na população idosa, especialmente em mulheres^(4,28,29)^.

Apesar do presente estudo não identificar correlação entre a idade e o SAPS 3, o risco de morte estimado pelo índice (14,6%) foi próximo ao observado na amostra (18,3%). Para os 11 idosos submetidos a tratamento conservador, a taxa de mortalidade durante a internação na UTI foi de 100%. Sobre isso, a gravidade desses idosos pode justificar a maior mortalidade observada pois, de acordo com os registros em prontuário, esses pacientes não tinham condições clínicas para o tratamento cirúrgico. Tratamento conservador, incapacidade de deambular antes da fratura e idade superior a 80 anos são condições que aumentam o risco de morte^(5)^, o que corrobora os achados da presente investigação, uma vez que 9 dos 11 idosos que não realizaram cirurgia para tratamento da lesão tinham mais de 80 anos de idade.

Os dados deste estudo mostraram número expressivo de idosos (61,3%) que apresentaram fratura de fêmur e que necessitaram de internação na UTI. Nesse sentido, vê-se a necessidade de aprimoramento das políticas de prevenção de queda e a garantia de uma vida saudável e da promoção de bem-estar da população idosa conforme preveem os Objetivos de Desenvolvimento Sustentável de Saúde e Bem-estar (ODS 3) para agenda 2030 da Organização das Nações Unidas (ONU)^(30)^. Ademais, esta pesquisa foi pioneira ao analisar a carga de trabalho de enfermagem mensurada pelo NAS na população idosa com fratura de fêmur, trazendo assim importantes contribuições que podem auxiliar no dimensionamento da equipe de enfermagem e na qualidade da assistência prestada aos pacientes na UTI.

Algumas limitações desta pesquisa devem ser consideradas. O tipo de estudo, caracterizado pela análise retrospectiva dos prontuários dos idosos, depende diretamente da qualidade e completude dos registros feitos pelos profissionais. Além disso, a pesquisa foi realizada em apenas um serviço, de atenção secundária e média complexidade, e este fato deve ser considerado na generalização externa dos achados. Vale ressaltar também que a instituição do presente estudo foi referenciada, a partir de 2017, para o atendimento de pacientes com doenças ortopédicas que necessitavam de cuidados pós-operatórios imediatos na UTI, o que pode justificar o aumento expressivo de admissões a partir deste ano.

Por fim, os resultados deste estudo permitiram compreender as características dos idosos que necessitam de tratamento intensivo em decorrência da fratura de fêmur e trazem importantes achados que podem subsidiar os profissionais da UTI, especialmente os enfermeiros, na elaboração de um plano de cuidados e na gestão de recursos humanos que atendam às necessidades desta população e garantam uma assistência segura. Além disso, os dados expressos nesta pesquisa mostram a necessidade do fortalecimento de políticas públicas voltadas às medidas preventivas de quedas, incluindo o reconhecimento dos fatores contribuintes para o evento, o estímulo à prática de atividade física, a implementação de intervenções personalizadas para idosos vulneráveis e com alto risco de cair e a orientação familiar, reduzindo assim a ocorrência deste tipo de lesão e necessidade de internação na UTI.

## CONCLUSÃO

Na análise da série histórica, o estudo evidenciou incremento das internações de idosos com fratura de fêmur na UTI a partir de 2017. Os resultados permitiram ainda concluir que pacientes do sexo feminino, com fratura distal de fêmur e que morreram na UTI, apresentaram idade mais elevada do que homens, pacientes com fraturas de diáfise ou proximal do fêmur e sobreviventes.

O estudo reforça a importância de os profissionais de saúde da UTI compreenderem as características e necessidades dos idosos com fratura de fêmur para estruturação do cuidado intensivo, implementação de protocolos assistenciais e capacitação da equipe para atendimento desta população tão específica. Além disso, sugere-se a realização de estudos como este em outros serviços e estados brasileiros para ampliar o conhecimento desta população e realizar futuros benchmarkings para melhoria da qualidade assistencial e, principalmente, das estratégias de prevenção das quedas, ainda tão frequentes em nossa população.
